# Histopathological Study of Host–Pathogen Interactions Between *Cordyceps javanica* PSUC002 and *Hypothenemus hampei*

**DOI:** 10.3390/jof11060423

**Published:** 2025-05-30

**Authors:** Sinlapachai Senarat, Peerasak Bunsap, Pisit Poolprasert, Anjaree Inchan, Natthawut Charoenphon, Peerapon Sornying, Narit Thaochan

**Affiliations:** 1 Division of Biological Science, Faculty of Science, Prince of Songkla University, Songkhla 90110, Thailand; sinlapachai.s@psu.ac.th; 2 Agricultural Innovation and Management Division (Pest Management), Faculty of Natural Resources, Prince of Songkla University, Songkhla 90110, Thailand; peerasakbunsap@gmail.com; 3 Department of Entomology, Faculty of Agriculture, Kasetsart University, Bangkok 10900, Thailand; fagrpspo@ku.ac.th; 4 Faculty of Medicine, Praboromarajchanok Institute, Ministry of Public Health, Nonthaburi 11000, Thailand; anjaree.in@gmail.com; 5 Department of Anatomy, Faculty of Medical Science, Naresuan University, Phitsanulok 65000, Thailand; natthawutch@nu.ac.th; 6 Department of Veterinary Science, Faculty of Veterinary Science, Prince of Songkla University, Songkhla 90110, Thailand; peerapon.s@psu.ac.th

**Keywords:** *Cordyceps javanica*, coffee berry borer, insect integument, conidia infestation

## Abstract

The use of entomopathogenic fungi (EPF), such as *Cordyceps javanica*, to reduce insect pest populations is gaining traction since it is an environmentally safe approach that can control many pests at different life stages. Here, we focus on the histopathology of the coffee berry borer, *Hypothenemus hampei*, infected by *C. javanica.* Morphological observation revealed that *C*. *javanica* conidia germinated within 12 h following inoculation according to light microscopic and ultrastructural levels. The fungus thoroughly penetrated the fat body and muscular tissue between 84 and 120 h post-inoculation. Transmission electron microscopy (TEM) confirmed the hyphal invasion of the cuticle at 12 h post-inoculation, with progressive tissue disruption and organelle degeneration, especially mitochondria and rough endoplasmic reticulum in adipocytes. All organelles were completely degenerated at 96 h post-inoculation. There was evidence of a connection between *C*. *javanica* activity and the coffee berry borer that might cause histopathological changes in the host defense against the pathogen, pointing to increased mortality and potential control of coffee berry borer in natural populations. Additionally, terminal deoxynucleotidyl transferase dUTP nick end labeling (TUNEL) confirmed that apoptotic cells were slightly increased in the adipose tissue and integument of the coffee berry borer. The ability of *C*. *javanica* to fatally infect the coffee berry borer suggests that it could be deployed as a biological control agent in the field.

## 1. Introduction

Between 2018 and 2023, global demand for coffee has remained strong; however, coffee production in many regions has declined due to climate change and increasing pest pressures, including infestations by the coffee berry borer (*Hypothenemus hampei*). The ideal conditions of temperature, humidity, rainfall and sunlight for growing coffee vary with the coffee type [1,2], and alterations in these environmental conditions can have a significant impact on coffee yield. These changes can also contribute to a rise in pest infestations [2,3]. The increasing temperatures and shifts in global rainfall patterns influenced pest proliferation in Mexico and Central America, adversely affecting coffee production [4]. The higher temperatures led to an upsurge in infestations by the coffee berry borer, *H. hampei*, causing damage to coffee crops globally [5,6]. These studies corroborated earlier findings that increasing global temperatures were linked to increased infestations of coffee beans by the coffee berry borer [7,8]. In Thailand, the *H. hampei* has damaged coffee crops on numerous occasions.

The coffee berry borer is a tiny beetle measuring 1.4–1.7 mm in length. It belongs to the family *Curculionidae* and the subfamily *Scolytinae*. It thrives in tropical and temperate regions [9,10] and is a severe problem for both small-scale and large-scale coffee producers, causing long-term damage [11]. It infests coffee fruits at all stages of development, from young green berries to mature red berries. Female beetles bore into coffee beans, potentially introducing fungi or bacteria, which start the infestation. Females lay 30–70 eggs, which then develop into chrysalises and adults. This leads to a decline in coffee quality and an increase in bean shedding before harvest, notably in Arabica and Robusta varieties [12]. Following infestation, bean shedding increases by 30–35% [10]. The remaining crop also grows more slowly. Furthermore, the affected coffee crop may realize a lower price, and the coffee will fail to meet international trade standards if more than 1.5% of the surface shows traces of infestation [13]. Various studies, including those that have focused on ways of controlling the coffee berry borer, include the development of pheromone trapping systems and biological control strategies using entomopathogenic fungi (EPF) [10,11]. There are over 700 species of EPF [14], and studies in pest management strategies have discussed the potential of *Beauveria bassiana*, *Metarhizium anisopliae*, and *Cordyceps fumosorosea* (currently named *C. javanica*) [15].

The physiological reactions of animals to infections can be understood by studying cellular and tissue alterations. Histopathology is an invaluable aid in understanding animal health and physical responses [16,17,18,19]. Histopathological research tells us that the fungus *M. anisopliae* can penetrate the midgut of the mosquito *Culex quinquefasciatus* in 48 h, causing the degeneration and death of intestinal absorptive cells [16]. This activity exemplifies the role of EPF in biological control. Both *B*. *bassiana* and *M*. *anisopliae* could infect the maize pest *Peregrinus maidis*, causing clear histopathological changes in muscles and organs [17]. A prior study reported histopathological changes in the skin and hemocytes of *Zeuzera pyrina* larvae within 3–4 days of exposure to *B*. *bassiana*, suggesting a physiological interaction between the fungus and its host [20]. These findings aligned with the work [18] that *M*. *anisopliae* could breach the skin and trigger degeneration in the cuticle-forming cells of the silk moth *Bombyx mori*, a mechanism of skin destruction driven by pathogenic fungi in other insects [14,21]. *C*. *fumosorosea* (formerly known as *Isaria fumosorosea*) is a fungus frequently encountered in various insect orders, including Diptera (mosquitoes, flies), Hemiptera (moths, aphids), and Lepidoptera (butterflies). The fungus is known for its pathogenic effects on these insects [22,23,24] and is increasingly utilized as a biological control agent against insect pests [25,26,27,28].

Although the traces of *C*. *fumosorosea* were isolated from the surface of the psyllid *Diaphorina citri* [29], research into the tissue-destructive capabilities of this fungus in insect pests has been somewhat limited. Previous studies have focused on mortality rates [30], enzyme activity [28], or fungal structure [28,31,32]. The production of hyphae by *C*. *fumosorosea* found that fungal hyphae formed a structure on the exposed host within 6 h and were observed, under transmission electron microscopy, to continue proliferating over 120 h [28]. Given these findings, the current research explores the pathogenic potential of *C*. *javanica* PSUC002 against the coffee berry borer by investigating the histopathological alteration of the coffee berry borer after exposure to *C*. *javanica* at light microscopic and ultrastructural levels. This study, describing the activity of *C*. *javanica* and its physiological response in the coffee berry borer, supports the broader use of EPF in future pest management.

## 2. Materials and Methods

### 2.1. Collection of Coffee Berry Borer Samples

Coffee berry borer specimens were gathered by examining coffee beans for signs of beetle infestation. Infested plant parts were collected and carefully placed in plastic containers that were transported to the laboratory for analysis. Pest management specialists from the Division of Agricultural Innovation and Management, Faculty of Natural Resources, Prince of Songkla University, separated beetles from plant material in the laboratory. Individual specimens were placed in a 5 cm diameter Petri dish and examined using a stereo microscope (Leica S8 APO with an apochromatic 8:1 zoom lens, Wetzlar, Germany). Following the methods described by Johnson et al. [33], this process enabled the identification of the specific beetles infesting the coffee plants.

### 2.2. Preparation of Insect Pathogenic Fungi

**Figure 1:**
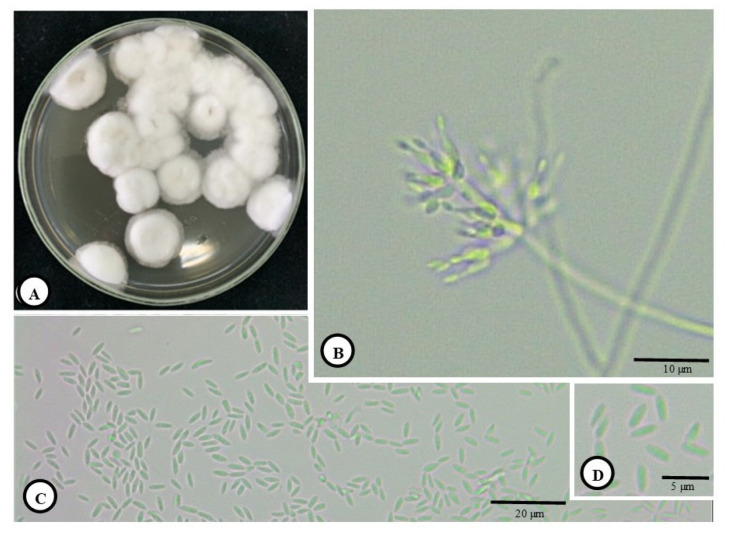
Morphology of *C. javanica*, (**A**): Colony of cultured *C*. *Javanica.* (**B**): Phialides formation of *C*. *Javanica* and (**C**,**D**): separated conidia spores.

The Pest Management main laboratory keeps EPF that affects various insects. *C*. *javanica* was cultured in slanted flasks and then transferred onto SDAY medium in Petri dishes, which were incubated in darkness at 28.0 ± 0.2 °C with a 12:12 h light/dark photoperiod for 14 days to ensure full spore formation (Figure 1A,B). Following the collection of fungal spores, a spore suspension with a density of roughly 1 ± 10^6^ spores per mL was prepared for further experimental use.

### 2.3. Infestation Mechanism of C. javanica on the Coffee Berry Borer

To explore how *C. javanica* penetrates the coffee berry borer, 320 adults of both sexes were exposed to the EPF. Each specimen was immersed in 10 mL of fungal spore suspension for 1 min. Subsequently, 50 mature specimens were placed in three Petri dishes (9 cm in diameter) lined with Whatman #1 filter paper soaked with sterilized distilled water. The beetles were housed in a laboratory temperature of 28.0 ± 0.2 °C under a 12-h light/dark photoperiod. There was also a control group of 10 specimens [34]. From 6 to 144 h post-inoculation, 20 individuals were sampled at 10 time points. At each sampling time, beetles were rapidly cooled using ice [35]. Their tissues were preserved in 100 µL of stabilizer in a 1.5 mL microcentrifuge tube for 36 h before being transferred to 70% ethanol and stored at 4.0 °C.

The mortality rate of beetles was recorded at each interval to determine the overall percent mortality. At each sample collection, anesthetized beetles were immersed in a preservative solution for detailed studies of the attack mechanism at the organelle, cellular, and tissue levels, including cell death. For this study, three samples were randomly selected for extensive structural analysis. The rest were employed to study tissue-level effects and cell death.

### 2.4. Histology and Histopathology

All coffee berry borer specimens were fixed in Davidson’s fixative for 24 h before being processed histologically. Following the methodologies described by investigators [36,37], tissue sections approximately 4 μm thick were produced and stained with hematoxylin and eosin (H&E) for structural and histopathological examination. This procedure also included computing the histological alteration index (HAI). To analyze the chemical composition and detect the presence of fungi in tissues, some slides were stained with Masson’s Trichrome, Periodic Acid-Schiff (PAS), and Grocott’s Methenamine Silver (GMS), following the guidelines of Bancroft and Gamble [36] and Dietrich and Krieger [38]. Observations were performed with a standard light microscope, and images were captured with a 3D Histech Pannoramic SCAN 150 (Pannoramic Scanner Software 150 1.14, Budapest, Hungary), utilizing 3DHISTECH’s slide scanning technology. Moreover, skin thickness measurements were randomly taken at six separate locations. The size of Malpighian cells and gastrointestinal nutrition absorption cells were quantified using ImageJ software 1.53 K application (NIH, Bethesda, MD, USA; available at https://imagej.nih.gov/ij/download.html, 1 April 2025) on three permanent slides per sample, for a total of thirty slides per experimental group.

### 2.5. Production and Measurement of Cuticle-Degrading Enzymes

We previously investigated the activity of cuticle-degrading enzymes, including chitinase and protease, from *C*. *javanica* [39]. Therefore, in the present study, only lipase activity was examined, following previous research [40], as lipases play a key role in degrading insect cuticular lipids during early fungal infection. Although we previously investigated protease and chitinase activities [39], lipase was selected for detailed analysis in this study due to its prominent role in host tissue colonization. To assess the lipase activity of *C. javanica*, we used 5 mm mycelial agar discs (n = 3) and transferred them onto a tributyrin agar base supplemented with 1% glycerol tributyrate. The discs were incubated at 25 °C for 5, 7, and 9 days, and their clears were also calculated according to prior published data [41]. Both colonized, and clear zones of cuticle-degrading enzymes were expressed as means ± SD.

### 2.6. TUNEL Analysis of Cell Death

The terminal deoxynucleotidyl transferase dUTP nick end labeling (TUNEL) technique was employed to detect and pinpoint cell death. This approach was applied to unstained slides from the tissue sections mentioned in the previous section, using the usual protocols outlined [42]. Subsequently, cell death was observed using a regular light microscope. Images of cells were taken for documentation, and maps depicting the distribution of cell death were created. The dead cell count was determined by specifying an area under 40× magnification. Using the ImageJ software, this count was based on three fixed slides from each sample for 30 permanent slides for the entire experimental set. Moreover, the density of cell death was evaluated using a semiquantitative analytical scale, with ‘−’ indicating no immunoreactivity, ‘+’ weak immunoreactivity, ‘++’ moderate immunoreactivity, and ‘+++’ strong immunoreactivity.

### 2.7. Ultrastructural Investigations

A small piece of the coffee berry borer was immediately pre-fixed in 2.5% glutaraldehyde in 0.1 M phosphate buffer (pH 7.4) for 24 h at 4 °C and then post-fixed with a post-fixative 1% osmium tetroxide (OsO_4_, 1% *v*/*v*) (Sigma Company, St. Louis, MO, USA) for 2 h. All samples were dehydrated in a series of acetone and embedded in Araldite^®^ (Sigma Company). Using a glass knife, semithin sections were cut and stained with toluidine blue to confirm the correct localization under a light microscope. Ultrathin sections were cut to a thickness of 60 to 70 nm in thickness by using an ultramicrotome (EM UC7, Leica, Inc., Wetzlar, Germany), mounted on grids and doubled-stained with uranyl acetate and lead citrate. Analysis of the corrected features and organelles of all specimens was performed with electron microscopy (Philips/FEI Tecnai G2 F20, FEI Co., Eindhoven, The Netherlands).

### 2.8. Statistical Analysis

The data acquired on skin thickness, Malpighian cell dimensions, gastrointestinal nutrition absorption cell sizes, and dead cell counts were initially summarized by calculating means and standard deviations. Subsequently, the aggregated data was statistically tested to identify significant differences among the experimental groups. This analysis was conducted using one-way ANOVA and two-way ANOVA in the IBM SPSS Statistics software, version 22.0 (IBM Corp., Armonk, NY, USA). To verify the reliability of the results, these tests were carried out with a confidence interval set at 95%.

## 3. Results

### 3.1. Morphology and Development of C. javanica and Penetration of Host Cuticle

**Figure 2:**
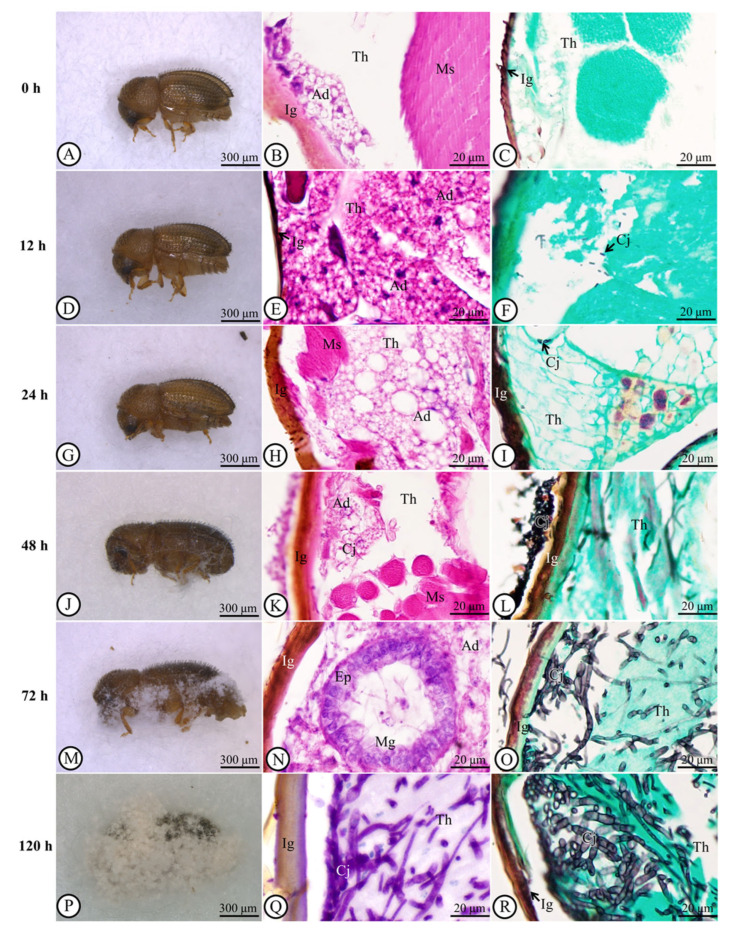
Histopathological development of *C. javanica* in a *H. hampei* host. (**A**): Adult *H. Hampei*. (**B**): Normal integument structure by H&E staining. (**C**): GMS stain performed on integument tissue. (**D**): At 12 h post-inoculation, hyphal invasion of the fat body underlying the cuticle stained with H&E (**E**) and GMS (**F**). (**G**): At 24 h post-inoculation, hyphal invasion of the fat body underlying the cuticle, stained with H&E (**H**) and GMS (**I**). (**J**): At 48 h post-inoculation, hyphal invasion of fat body underlying the cuticle, stained with H&E (**K**) and GMS (**L**). (**M**): At 72 h post-inoculation, hyphal invasion of fat body underlying the cuticle, stained with H&E (**N**) and GMS (**O**). (**P**): At 120 h post-inoculation, hyphal invasion of fat body covering the cuticle, stained with H&E (**Q**) and GMS (**R**). Staining methods: H&E = hematoxylin and eosin, GMS = Grocott’s methenamine silver stain. Abbreviations: Ad = adipose tissue, Cj = *C. javanica*, Ep = epithelium, h = hour, Ig = integument, Mg = midgut, Ms = muscle, Th = thorax.

The general morphologies of the colony, phialides and conidia (separated) of cultured *C*. *javanica* are presented in Figure 1A–D, where Figure 1A shows the colony morphology, Figure 1B presents the phialides and Figure 1C,D displays the conidia. The progression of the spread of the fungus on the coffee berry borer was also demonstrated from 0 to 120 h (Figure 2A–R). At 0 h, there was no visible sign of the fungal growth on the coffee berry borer (Figure 2A–C). Hyphal bodies were initially detected in the cuticle at 12 h and became more prominent at 48 h post-inoculation (Figure 2D–L). As the infection progressed, hyphae penetrated the cuticle, muscles, and midgut between 72 and 120 h post-inoculation (Figure 2M–R). The fungal hyphae colonized the host’s external morphology and penetrated epithelial cells into the gut lumen, eventually colonizing the adipose tissue (Figure 2R).

### 3.2. Fungal Spread in the Cuticle and Post-Inoculation Cuticle Thickness of Host

**Figure 3:**
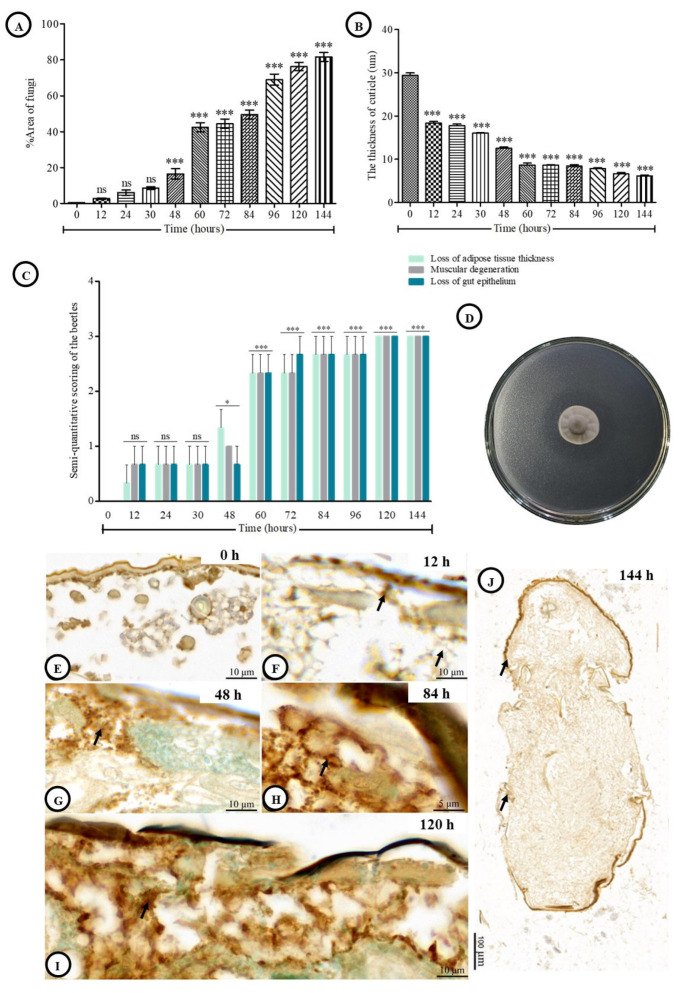
The bar graph and Light microscope observation of apoptotic cells (arrows) in adult *H. hampei* specimens. (**A**): A comparison of percentage areas of fungal infection of the control (0 h) and at various times after inoculation with *C. javanica*. Values are presented as means ± SE. Statistically highly significant (*** *p* < 0.001). (**B**): The thickness of the cuticle of *H. hampei* after inoculation with *C. javanica*. Values are presented as means ± SE. Statistically highly significant (*** *p* < 0.001). (**C**): Histopathological alteration indexes (HAI) of adult *H. hampei* specimens inoculated with *C. javanica*. Data compare the loss of adipose tissue thickness, muscular degeneration and loss of gut epithelium. Values are presented as means ± SE. Statistically highly significant (*** *p* < 0.001). (**D**): The lipase production in mycelial agar discs. (**E**–**J**): The apoptotic cells (arrows) in adult *H. hampei* specimens inoculated with *C. javanica* at different post-inoculation times: 0 h (**E**), 12 h (**F**), 48 h (**G**), 84 h (**H**), 120 h (**I**) and 144 h (**J**). Statistical significance: * *p* < 0.05 and ns = non-significant.

GMS revealed the presence of *C*. *javanica* from 12 to 144 h post-inoculation. The fungal invasion had significantly increased when compared with the presence of the fungus at 0 h post-inoculation (Figure 2A–R). At 48, 60, 72, 84, 96, 120 and 144 h post-inoculation, the areas of fungal spread were 16.52 ± 2.92%, 42.45 ± 2.44%, 44.44 ± 2.64%, 49.47 ± 2.54%, 68.94 ± 3.15%, 76.27 ± 2.33%, and 81.64 ± 2.49% (*p* < 0.001), respectively (Figure 3A). Mean cuticle thickness significantly decreased (*p* < 0.001) between 12 and 144 h compared to the initial measurement of the cuticle at 0 h post-inoculation. At 12 h post-inoculation, cuticle thickness (18.60 ± 0.47 µm) began to reduce significantly to 14.34 ± 0.55 µm at 24 h, 13.17 ± 0.14 µm at 30 h, 13.05 ± 0.48 µm at 48 h, 8.79 ± 0.29 µm at 60 h, 8.39 ± 0.31 µm at 72 h, 8.38 ± 0.28 µm at 84 h, 8.07 ± 0.37 µm at 96 h, 7.41 ± 0.25 µm at 120 h, and 6.07 ± 0.40 µm at 144 h (Figure 3B).

### 3.3. Histological Alteration Indexes (HAI)

To assess the extent of tissue destruction caused by fungal infection, the Host Area Index (HAI) was calculated based on three parameters: loss of adipose tissue thickness, muscular degeneration, and epithelial loss in the gut (Figure 3C). All parameters showed a significant increase in damage compared to 0 h post-inoculation. The highest levels of destruction for each parameter were recorded at 144 h post-inoculation, with all reaching an HAI score of 3. Overall, the HAI values exceeded the established severity threshold, indicating substantial tissue damage due to infection (Figure 3C).

### 3.4. The Determination of Lipase Activity

Lipase production by *C*. *javanica* was clearly observed on tributyrin agar plates (Figure 3D). The average diameter of the fungal colony was 2.93 ± 0.25 mm, while the surrounding clear zone measured 5.25 ± 0.92 mm, indicating active lipid hydrolysis. The larger clear zone relative to the colony demonstrates extracellular secretion and diffusion of lipase into the medium, which is crucial for cuticle degradation during fungal infection.

### 3.5. Semiquantitative Analytical Score of TUNEL-Positive Cells and PAS Reaction

At 0 h post-inoculation, undamaged tissue exhibited only rare nuclei of apoptotic cells, indicating no immunoreactivity (Figure 3E). At 12 h post-inoculation, the percentage increased slightly to 12.3 ± 1.4% (Figure 3F and Table 1). By 48 h post-inoculation, apoptotic cells had increased significantly to 43.5 ± 2.8% (Figure 3G). The percentage continued to rise, reaching peak levels between 84 and 144 h post-inoculation, with values ranging from 68.9 ± 3.2% to 76.2 ± 3.4% (Figure 3H–J and Table 1). It should be noted that the fungus eventually covered the entire body of the host (Figure 3J).

### 3.6. Ultrastructural Observation

#### 3.6.1. Integumental Ultrastructure

**Figure 4:**
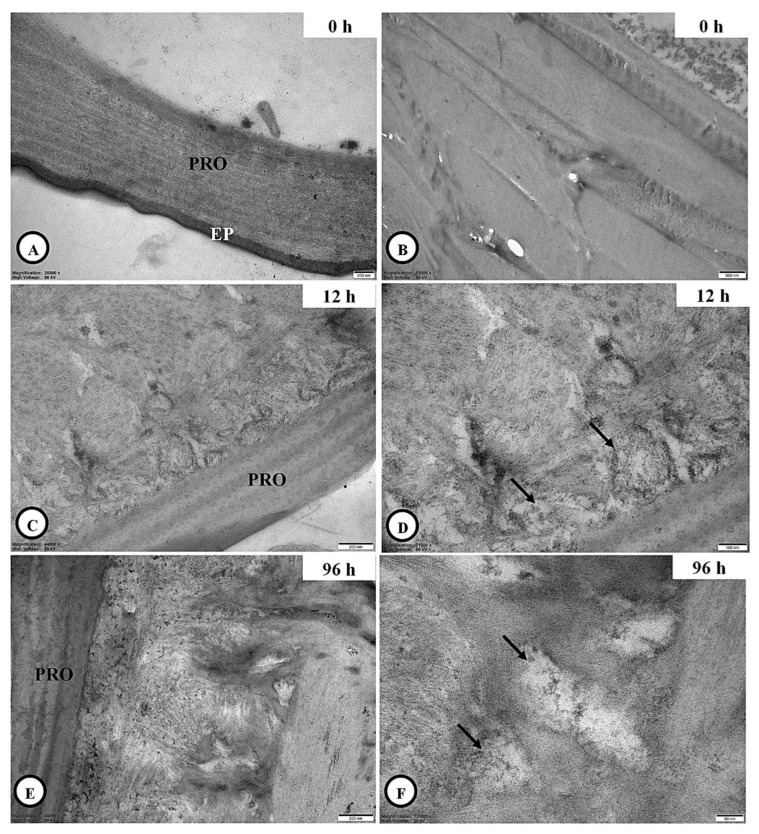
Ultrastructural integuments of adult *H. hampei* specimens after exposure to *C. javanica*. (**A**,**B**): A clear observation of integument having the procuticle (PRO) and epicuticle (EP) at 0 h post-inoculation. (**C**,**D**): A few penetrations of hyphae with an amorphous and electron-lucent (arrows) of *C. javanica* throughout the procuticle (PRO) at 12 h post-inoculation. (**E**,**F**): The well penetrations of germination and hyphal structure of *C. javanica* at 96 h post-inoculation.

To investigate ultrastructural alterations and fungal penetration in the coffee berry borer cuticle, transmission electron microscopy (TEM) was used to observe morphological changes at various post-inoculation intervals (Figure 4). At 0 h post-inoculation, the coffee berry borer cuticle displayed a normal structure, with clearly defined epicuticle and procuticle layers and no signs of fungal invasion (Figure 4A,B). By 12 h post-inoculation, fungal hyphae of *C. javanica* had begun to vertically penetrate the epicuticle and procuticle, appearing as amorphous, electron-lucent structures, indicating the initial stages of infection (Figure 4C,D). At 96 h post-inoculation, infection had progressed substantially; germinated hyphae exhibited larger vacuoles and deeper invasion into host tissues, highlighting significant host tissue disruption (Figure 4E,F).

#### 3.6.2. Adipocyte Ultrastructure

**Figure 5:**
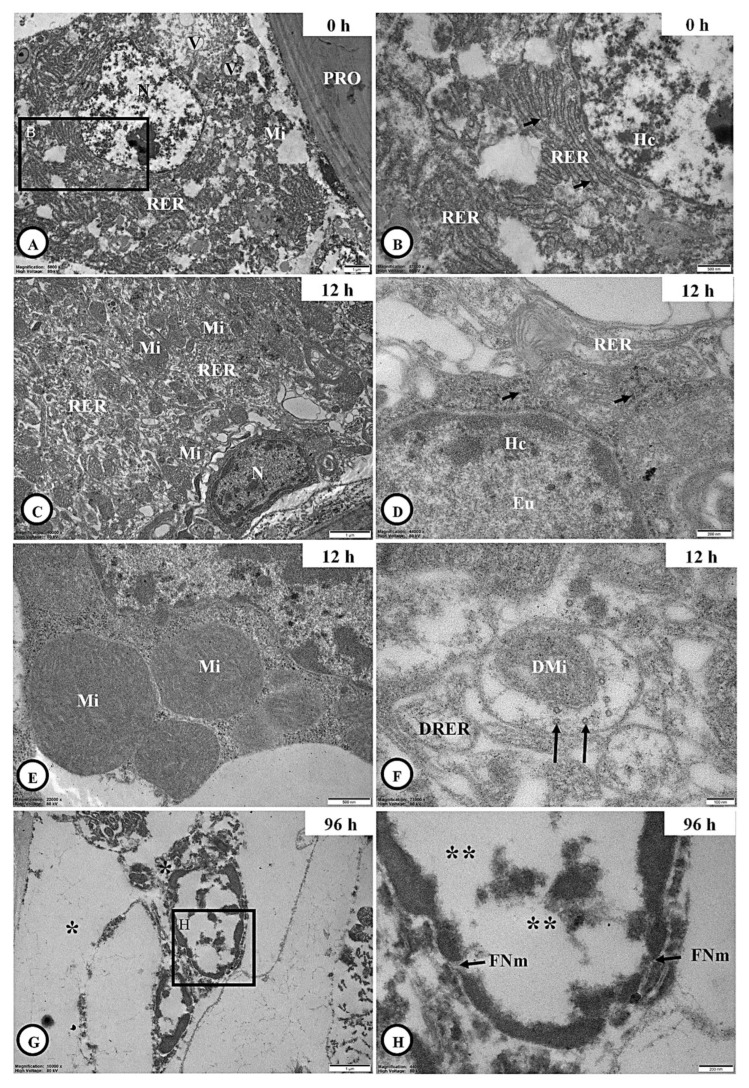
Ultrastructural adipocyte of adult *H. hampei* specimens after exposure with *C. javanica*. (**A**,**B**): The adipocyte at 0 h post-inoculation with the occurrence of a large central nucleus and the well-development of rough endoplasmic reticulum (RER) together with the ribosomes (arrows), mitochondria (Mi) and vacuoles (V). (**C**–**E**): The adipocyte at 12 h post-inoculation with the similar previous post-inoculation was observed; however, the RER, mitochondria (Mi) and ribosomes (arrows) were degenerated in some areas of this cell (**F**). (**G**): The adipocyte without the organelles above (single asterisk) at 96 h post-inoculation, whereas its nucleus with the fragmented nuclear membrane (FNm) and euchromatic necrosis (double asterisks) were identified (**H**). Abbreviations: Eu = euchromatin, Hc = heterochromatin, PRO = procuticle.

At 0 h post-inoculation, adipocytes were commonly located near the integument (Figure 5A). These cells were characterized by the presence of a large central nucleus, abundant rough endoplasmic reticulum (RER), mitochondria, and storage vacuoles. At higher magnification, prominent lysosomes surrounding the RER were also observed (Figure 5B). At 12 h post-inoculation, the cytoplasmic structure of the adipocytes remained similar to that at 0 h post-inoculation (Figure 5C–E); however, partial degeneration of RER, mitochondria and lysosomes was evident in some areas (Figure 5F). By 96 h post-inoculation, most of these organelles were no longer visible, and additional signs of cellular damage, including fragmented nuclear membranes and euchromatic necrosis within the nucleus, were observed (Figure 5G,H).

#### 3.6.3. Skeletal Muscle Ultrastructure

**Figure 6:**
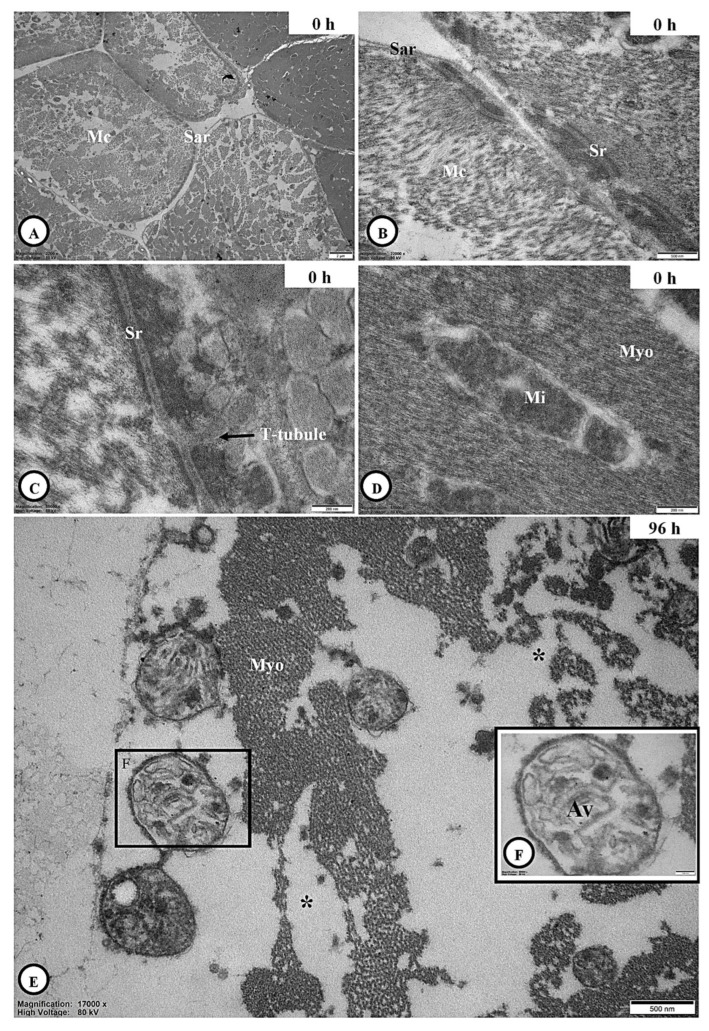
Ultrastructural muscle cells of adult *H. hampei* specimens after exposure with *C. javanica*. (**A**): The muscle cell (Mc) surrounding the sarcolemma (Sar) at 0 h post-inoculation was described, whereas its sarcoplasmic reticulum (Sr) was corrected (**B**). (**C**,**D**): High magnification showed the transverse (T-tubules are linked with the sarcoplasmic reticulum (Sr) and the mitochondria (Mi)-associated myofibrils (Myo) in the muscle cells. (**E**,**F**): The appearance of necrosis of myofibrils (*) with several autophagic vacuoles (Av) was observed at 96 h post-inoculation.

Several muscle cells of the coffee berry borer at 0 h post-inoculation were examined in cross-section and found to be enclosed by a well-defined sarcolemma (Figure 6A). At higher magnification, the sarcoplasmic reticulum was observed in proximity to the mitochondria (Figure 6B), while transverse (t)-tubules, which are invaginations of the muscle cell membrane, were clearly visible (Figure 6C). In addition, mitochondria-associated myofibrils were evident (Figure 6D). At 96 h post-inoculation, signs of muscle degeneration became apparent, including necrotic myofibrils and the presence of autophagic vacuoles (Figure 6E,F).

## 4. Discussion

The potential of the EPF *B*. *bassiana* and *M. anisopliae* to control the coffee berry borer has been addressed extensively in laboratory conditions [43,44,45,46]. This study is the first attempt to apply *C*. *javanica* to control the coffee berry borer in Thailand. It was found that *C*. *javanica* had penetrated the integument of the coffee berry borer at 12 h and had entered the body within 24 h of exposure. Lesions appeared at 12 h and increased in size, accompanied by cell death and integument weakening. Regarding fungal accumulation in the beetle cuticle, fungal hyphae are attached to all body regions of the host, as previously reported by Boucias et al. (1988) [47]. The histopathological efficacy of *C. javanica* against the coffee berry borer was similar to that of other EPF that infected the coffee berry borer [46], the mosquitoes *Ades agypti* [48], the termite *Odontotermes obesus* [49], and the green bug *Nezara viridula* [50]. Hence, we recommend that *C. javanica* is the augmentative significance of a biological control agent to control the coffee berry borer.

Typically, *C. javanica* conidia germinated within 12 h following inoculation. The germ tube lengthened slowly. Two possible forms of fungal activity might happen during germination. Initially, the distal end of the conidial germ tube can form a specialized infection needle that penetrates the cuticle. Furthermore, the germ tubes of penetrating conidia produce hyphae that spread across the integument until a suitable penetration site is located when a specialized infection nodule forms at the tips of the hyphae, which penetrates the cuticle. Conidia and appressoria had slimy surfaces [48,51]. Most investigations of insects exposed to EPF have demonstrated infections of the epithelial loss of the midgut, which may differ from the case of the coffee berry borer. The TUNEL assay revealed marginal increases in the numbers of apoptotic cells in coffee berry borer adipose tissue and the integument. Our data, therefore, increased further information about the *C. javanica* infection process via the histopathological evidence in the coffee berry borer, indicating its potential application in site-specific management of the coffee field.

TEM revealed hyphae penetrating the epicuticle and procuticle, along with electron-lucent areas indicative of tissue degradation. By 96 h post-inoculation, structural collapse of the integument was evident. These findings align with the reported infection mechanisms of other entomopathogenic fungi, which utilize enzymatic and mechanical strategies to breach the cuticle [46]. We hence confirmed that *C*. *javanica* releases digestive enzymes such as lipase (present study), chitinase, and protease [39] that could break down chitin, lipids, and protein as similarly reported in other [52,53,54]. At this point, the infected beetle broke down and lost coordination in its locomotor responses. Similar pathogenic evidence has been encountered across different insects and arthropods infected with EPF [47,48,49,50,55,56,57]. Similar temporal patterns of fungal penetration have been described for *C. fumosorosea* and *M. anisopliae* [28,48]. Strain-specific pathogenicity supported the high virulence of *C. javanica* PSUC002; the use of both LM and TEM hence confirms true tissue invasion, distinguishing it from surface colonization [29].

We revealed that adipocyte degeneration began at 12 h post-inoculation and was pronounced 96 h post-inoculation. LM showed reduced tissue thickness and cellular disintegration, while TUNEL assays indicated a progressive increase in apoptosis. TEM confirmed early organelle damage, particularly to mitochondria, rough endoplasmic reticulum (RER), and lysosomes and complete organelle loss by 96 h post-inoculation. This degeneration is consistent with prior studies on entomopathogenic fungi, which reported fat body damage and metabolic impairment in infected insects [17,50]. The nuclear abnormalities observed are characteristic of fungal-induced apoptotic pathways [25], suggesting that *C*. *javanica* disrupts energy metabolism and immune functions in the host.

LM identified disorganized muscle fibers and elevated histopathological scores, while TEM clearly revealed necrotic myofibrils and autophagic vacuoles. In healthy tissue, sarcomeres, mitochondria, and sarcoplasmic reticulum were intact; however, these features were severely compromised by post-infection, indicating impaired muscular function. Comparable muscle degeneration has been observed in insects infected with *M*. *anisopliae* and *B*. *bassiana*, including the formation of autophagic vacuoles [47,48,50]. These consistent outcomes across taxa emphasize muscle degeneration as a hallmark of fungal pathogenesis, contributing to paralysis and mortality in infected hosts.

## 5. Conclusions

The histological and ultrastructural evidence indicated that the coffee berry borer is susceptible to infection by *C*. *javanica*. The complete developmental cycle of the pathogen, which begins with penetration of the integument at about 12 h post-inoculation, parallels that of this fungus in other tissues. The efficacy of this pathogen in parasitizing the coffee berry borer suggests its potential as a biological control agent in the field.

## Figures and Tables

**Table 1:** Semiquantitative analytical scores of TUNEL-positive cell numbers at each time point.

Parameter	Times						
	0	12	48	84	96	120	144
TUNEL-positive cells	−	+	++	+++	+++	+++	+++

Semiquantitative analytical scale: ‘−’ indicates no immunoreactivity, ‘+’ weak, ‘++’ moderate, and ‘+++’ strong immunoreactivity.

## Data Availability

The data presented in this study are available on request from the corresponding author.
